# Attentional load impacts multisensory integration, without leading to spatial processing asymmetries

**DOI:** 10.1038/s41598-025-95717-0

**Published:** 2025-05-09

**Authors:** M. S. Saccani, G. Contemori, F. Del Popolo Cristaldi, M. Bonato

**Affiliations:** 1 Padova Neuroscience Centre, University of Padua, Via Orus 2, 35129 Padua, Italy; 2 Department of General Psychology, University of Padua, Via Venezia 8, 35131 Padua, Italy

**Keywords:** Spatial processing, Multisensory integration, Attentional load, Unimpaired cognitive system, Neuroscience, Auditory system, Sensory processing, Visual system, Human behaviour

## Abstract

The present study examined whether spatial processing in the unimpaired cognitive system is influenced by attentional load during multitasking. More specifically, it tested the hypothesis that high attentional load would induce spatial processing asymmetries in the form of a rightward attentional bias. We conducted two separate experiments on healthy adults (n = 101 and n = 98) by using web-based data collections. We capitalized on a condition of perceptual uncertainty to investigate the presence of these spatial asymmetries which cannot be easily detected under regular perceptual conditions. More specifically, we employed a primary audiovisual integration task, which involved presenting stimuli capable of eliciting the sound-induced flash illusion (i.e., task-relevant flashes accompanied by an incongruent number of sounds) on either the left or right side of the screen. This task enabled us to investigate audiovisual integration, but also indirectly provided an opportunity to sensitively explore spatial processing within a highly complex context. In Experiment 1, attentional load was increased by presenting stimuli to be retained *before* the audiovisual integration task (i.e., “offline” attentional load manipulation). Differently, in Experiment 2, attentional load was increased by having participants to perform visual discrimination *during* the audiovisual integration task (i.e., “online” attentional load manipulation). Attentional load was increased in a different way within each experiment to test the idea that more demanding tasks, albeit of different nature, would have similarly modulated performance. In both experiments, we replicated the increase of sound-induced flash illusion under high attentional load, which challenges the notion of an early and pre-attentive onset of the illusion. However, this effect was identical for left- and right-sided flashes, which speaks against the existence of load-induced spatial processing asymmetries in the unimpaired cognitive system. Given that both experiments yielded similar results, quantitative aspects of attentional engagement rather than the nature of the attentional resources involved seem to play a critical role.

## Introduction

### Theoretical background

While we navigate in the environment, we frequently encounter a vast amount of visual information that surpasses our processing capacity. Luckily enough, in numerous situations, this is not a problem as humans are equipped with very efficient spatial processing skills, to the extent that we continuously use them without even realizing how pervasive their presence is. Spatial processing is implemented by selective attention mechanisms and allows us to effectively deal with this broad flow of visual information in a seemingly effortless and highly efficient way^1^. More specifically, thanks to these selective attention mechanisms, under normal circumstances we can process the most salient or potentially important stimuli within a specific location in space, while ignoring all others^1–3^. Yet, spatial processing efficiency seems to be negatively affected by levels of attentional load. This is proved by the emergence of spatial processing modulations or biases, when spatial processing is made more challenging through additional demands^4–16^. This influence is commonly investigated in experimental contexts with a multi- or dual-tasking approach, for instance by coupling a primary task that requires spatial processing with concurrent secondary tasks that lead attentional load to increase, as all the tasks must be completed simultaneously^17^.

An ongoing debate within the research field of load-induced spatial processing modulations pertains to the existence of *asymmetric* modulations. These modulations have been extensively examined in neurological populations, where they are often prominent, and in healthy populations, where they tend to be more subtle. With respect to neurological populations, it is crucial to mention that spatial processing deficits for the contralesional hemispace, such as extinction and neglect, are common consequences of unilateral brain damage to many cortical and subcortical brain regions ^18,19^. According to the literature, load-induced spatial processing asymmetries can emerge very clearly in brain-damaged patients. They often show some degree of ipsilesional attentional bias which can be further exacerbated, sometimes dramatically, by multitasking ^4,11–16^. When it comes to healthy populations, the issue at stake is more subtle and focused on whether spatial processing is perfectly symmetric or whether some differences are present between left- and right-hemispace processing. This question holds particular significance for understanding the properties of the unimpaired cognitive system, but also for the interpretation of clinical data: understanding the performance of the “baseline system” is essential for quantifying the extent of impairment in clinical cases. Albeit much smaller in amplitude, load-induced spatial processing asymmetries have been reported also in healthy adults. They tend to show either a reduction of the typical leftward attentional bias called pseudoneglect or even a rightward attentional bias, that consists in a disadvantage for the processing of left-sided stimuli compared to right-sided stimuli during multitasking^4–10^. For example, Peers and colleagues^4^ asked participants to perform a visual detection task in which an array of six letters was briefly presented around a central fixation point, firstly in isolation and then while simultaneously engaging in a spatial or non-spatial auditory discrimination task. In the low attentional load condition, performance in the visual detection task was symmetrical. Differently, in the high attentional load condition, when the two tasks had to be performed together, a significant rightward attentional bias emerged, consisting of reduced accuracy in reporting left-sided but not right-sided letters^4^. Similarly, Naert and colleagues^8^ required participants to perform a visual detection task of lateralized dots coupled with a working memory task (i.e., rehearsing short vs long stimuli sequences). Also in this case, in the low attentional load condition, performance in the visual detection task was symmetrical. In contrast, in the high attentional load condition, when long sequences of stimuli had to be remembered, a significant rightward attentional bias emerged, consisting of increased response times in detecting left-sided but not right-sided dots^8,9^. Moreover, Pérez and colleagues^5,6^ paired a temporal order judgement task with an attentional blink task and replicated the attentional load level effect. Participants were presented with a visual target (T1 of the attentional blink task) and after a variable delay (280 or 1030 ms) they were shown a pair of lateralized stimuli (T2 of the attentional blink task) that had to be temporally ordered. In the low attentional load condition, when T1 was ignored or the lag between T1 and T2 was long, a leftward attentional bias^5^ or no bias^6^ emerged. In contrast, in the high attentional load condition, when T1 had to be processed plus the lag between T1 and T2 was short, an attention shift towards the right was found: left-sided stimuli were perceived as appearing simultaneously with right-sided stimuli even when they were in fact presented few milliseconds prior to them^5,6^. Finally, similar results were replicated also in more ecological contexts like a driving simulator^7^ or a virtual maze^10^. However, the abovementioned studies reporting load-induced spatial processing asymmetries in healthy adults are characterized by small sample sizes, which limit the generalizability of their findings (Peers et al.^4^: Experiment 1 and Experiment 2, n = 11; Naert et al.^8^: Experiment 1 and Experiment 2, n = 20; Pérez et al.^5^: Experiment 1 and Experiment 2, n = 14; Pérez et al.^6^: n = 20; Benedetto et al.^7^: n = 15; Bartlett et al.^10^: n = 30). Moreover, some studies provided evidence supporting the *absence* of load-induced spatial processing asymmetries in healthy adults. For example, Bonato and colleagues^11,12,14,15^ tested right-hemisphere damaged patients and left-hemisphere damaged patients with a dual-task paradigm. The primary task involved detecting the appearance of lateralized visual targets and was completed in isolation or together with a concurrent secondary task that required categorizing visual or auditory stimuli. The dual-task condition (i.e., high attentional load condition), reliably detected contralesional omissions in the majority of patients, regardless on the specific nature of the dual-tasking requirements (e.g. visual or auditory). Still, despite being highly sensitive with both right- and left-hemisphere damaged patients, it systematically failed in revealing any significant rightward attentional bias in neurologically intact matched control participants^11,12,14,15^. This lack of bias was observed even when the duration of the lateralized visual targets was extremely brief (i.e., 50 ms) and their size was reduced from 0.8° to 0.3°^14^. Similarly, Russel and colleagues^16^ asked a group of right-hemisphere damaged patients to complete a visual discrimination task that varied in difficulty and was combined with an attentional blink task and the control group of neurologically intact matched control participants didn’t show any effect of attentional load level^16^. The absence of load-induced spatial processing asymmetries observed in control participants could be attributed to several factors. Firstly, patients may have more prominent general processing limitations compared to matched controls. Additionally, they may experience accelerated declines in efficiency as the task progresses. More importantly, patients may exhibit more pronounced spatial processing difficulties. As a result, dual-tasks that are challenging enough to increase attentional load during spatial processing in patients, while also being optimized in order not to result too complicated, are not ideal to modulate performance in control participants. Therefore, the absence of load-induced spatial processing asymmetries in control participants shouldn’t be interpreted as the absence of such modulations in the unimpaired cognitive system. Instead, such an absence could simply be the result of a ceiling effect (Bonato et al.^10^: controls accuracy in all conditions > 97.5%; Bonato et al.^15^: controls accuracy in all conditions = 100%; Russell et al.^16^: controls accuracy in all conditions > 80%; Bonato^14^: controls accuracy in all conditions = 100%; Blini et al.^11^: control accuracy in all conditions > 98%). Still, even if we were to ignore those manipulations which were primarily devised for testing neurological patients some studies which specifically tailored the task for healthy participants reported absence of load-induced spatial processing asymmetries. For example, Lisi and colleagues^20^ used a task similar to the one employed by Bonato and colleagues^12^ to test exclusively healthy adults’ performance. In this case, to avoid ceiling effects, during the primary task, lateralized visual targets were masked. Additionally, during the secondary task, it was required to categorize not only visual *or* auditory stimuli, but also visual *and* auditory stimuli together. Despite that, the interaction between attentional load level and target side did not reach significance. Another study exclusively focused on healthy adults’ performance was conducted by Dodds and colleagues^21^ utilizing the same task as Peers and colleagues^4^. This task proved to be sufficiently sensitive in detecting subtle spatial processing modulations in healthy adults, as indexed by a significant effect of time-on-task on spatial processing. Specifically, an attention shift towards the right was observed at the end of the task, but not at the beginning^21^. Despite that, the attention shift was not modulated by attentional load level, failing to replicate the previous result^21^.

Several explanations can account for the presence of load-induced spatial processing asymmetries. The interhemispheric competition theory by Kinsbourne, an early and influential model of visuospatial attention, suggests that each hemisphere generates its own “contralateral vector of attention”, with the two vectors competing against each other. The hemisphere with greater activation ultimately dominates, leading to a shift in attentional allocation toward the contralateral space^22–24^. Other theories of visuospatial attention differentiate between goal-driven attention (voluntarily directed toward a specific goal), and stimulus-driven attention (automatically triggered by salient or unexpected environmental changes). These two attentional processes are thought to be different not only functionally but also anatomically, engaging two distinct neural networks. The bilateral dorsal network, with the intraparietal sulcus (IPS) as a key node, is thought to be involved in goal-driven behaviours, while the right-lateralized ventral network, with the temporo-parietal junction (TPJ) as a crucial node, is thought to play a central role in stimulus-driven behaviours and in the reorienting of attention^25–27^. Interestingly, while bilateral IPS activity strongly correlates with increases in attentional load, this rise in demands is accompanied by a suppression of right TPJ activity, perhaps to prevent interference from task-irrelevant sources during the execution of attention demanding tasks^28^. Consistent with Kinsbourne’s theory, the resulting interhemispheric imbalance may ultimately trigger a rightward shift in attentional allocation^9,29^. However, the mechanism underlying load-induced spatial processing asymmetries remains to be clarified.

Given the mixed findings previously outlined, it becomes increasingly important to clarify whether spatial processing can be asymmetrically influenced by levels of attentional load during multitasking in the unimpaired cognitive system. However, investigating the performance of healthy adults necessitates the use of a sufficiently challenging task to avoid ceiling effects. To address this issue, we adopted an experimental design characterized by perceptual uncertainty which was generated through the use of lateralized multisensory stimuli. More specifically, we capitalized on an audiovisual integration illusion known as the “sound-induced flash illusion” (SIFI), which occurs during the simultaneous presentation of different numbers of visual flashes and auditory sounds. Participants’ accuracy in reporting the number of perceived flashes is higher when the number of flashes and sounds is congruent, but it drops when they are incongruent, as sounds influence flashes perception in an illusory way^30,31^. Specifically, when one flash is combined with two sounds, it can lead to the splitting of the visual percept, causing participants to report seeing two flashes (i.e., fission illusion). On the other hand, when two flashes are combined with one sound, it can induce the merging of visual percepts, leading participants to report seeing only one flash (i.e., fusion illusion)^30–32^. We reasoned that such context of perceptual uncertainty would be optimal for increasing task complexity, thereby enhancing sensitivity to detecting potential, rather subtle, spatial asymmetries which might not easily emerge under less demanding perceptual conditions.

The SIFI is highly robust and has been replicated in numerous studies, even with variations in different parameters^33,34^. Although SIFI has been associated with brain activity occurring 35–65 ms after the flash onset^35^ and activity in the primary visual cortex^36,37^, substantial evidence also suggests that it can be modulated by higher-level, top-down attentional factors^33,34^. For example, Michail and colleagues^38,39^ reported that the illusion generation is reliably influenced by concurrent task demands. They modulated attentional load exposing participants to stimuli eliciting the SIFI, while completing a concurrent n-back task. Results showed that high attentional load led to an increase in the rate of illusions^38^, associated with the engagement of top-down theta and beta frequency bands^39^. While the vast majority of SIFI experiments used central flashes in our experiment we decided for their lateralized presentation. It has been described that the probability of perceiving the illusion increases when the visual stimuli’s eccentricity is greater, possibly because audiovisual integration is more efficient in the periphery compared to the fovea^40,41^. Also the presence of lateralized stimuli therefore seems to be optimal for increasing task sensitivity and its potential for detecting spatial asymmetries. Moreover, in the absence of attentional load, there seem to be no differences in the rate of illusion perception when stimuli eccentricity is increased toward the right or the left side of space^42^.

Notably, while our manipulation is novel, robust load-induced spatial processing asymmetries have been already described to reliably emerge when using multisensory stimuli. In a study by Eramudugolla and colleagues^43^ participants were exposed to sequences of simultaneous but spatially separated auditory and visual stimuli, while they also had to discriminate centrally presented salient (i.e., high attentional load condition) or not salient (i.e., low attentional load condition) visual patterns. After this exposure, participants showed the ventriloquist aftereffect, that is a long-lasting shift in the localization of auditory stimuli, toward the direction of the visual stimuli presented during the exposure period. Crucially, the aftereffect was significantly enhanced after experiencing high attentional load, but only when the sound localization was shifted toward the right side of space and only for sounds on the right side of space. This result was likely due to a rightward attentional bias that emerged during exposure to salient visual patterns^43^.

### Aim and hypothesis

As thoroughly discussed before, spatial processing efficiency appears to be negatively affected by levels of attentional load. This phenomenon is well-documented in neurological populations, where load-induced spatial processing asymmetries are clearly evident. In healthy populations, however, this effect is more subtle and can potentially be masked by ceiling effects.

In order to clarify the existence of load-induced spatial processing asymmetries in the unimpaired cognitive system we conducted two separate experiments employing a computer-based dual-tasking approach with healthy adults. In both experiments, we utilized a primary audiovisual integration task that involved presenting stimuli capable of eliciting the SIFI on either the left or the right side of the screen. This task not only allowed to investigate audiovisual integration, but also indirectly provided an opportunity to investigate spatial processing under conditions of perceptual uncertainty. We reasoned that such context of perceptual uncertainty would be ideal for increasing task complexity, thereby enhancing sensitivity to subtle spatial asymmetries that might remain undetected in less challenging settings. Depending on the experiment, the primary task was paired with different secondary tasks requiring additional processing. In Experiment 1, the primary task was combined with a concurrent secondary verbal or spatial working memory task that enabled us to manipulate attentional load *offline* (i.e., the stimuli used to vary load were presented *before* the lateralized audiovisual integration stimuli). In Experiment 2, the primary task was instead combined with a concurrent secondary visual discrimination task that allowed us to manipulate attentional load *online* (i.e., the stimuli used to vary load were presented *simultaneously* with the lateralized audiovisual integration stimuli). Under the bold assumption that the quantity of attentional resources, rather than their nature, plays a critical role, we decided to employ secondary tasks which were different in nature but always challenging. We considered it important to evaluate the effects of different secondary tasks (i.e., offline/online tasks, working memory/visual discrimination tasks, and–within the working memory task–verbal/spatial stimuli), as it was not possible to establish a priori which type of attentional load variation would be more effective. Moreover, relying on a single type of manipulation could have raised concerns that any effects, if present, might stem from the specific nature of the secondary task (e.g., cognitive vs. perceptual or visual vs. auditory). A noteworthy feature of both experiments was the web-based data collection, which facilitated the participation of a large number of participants compared with previous research in the field.

We expected, across both experimental designs, (H1) SIFI to emerge with incongruent stimuli as the result of failed audiovisual integration. Additionally, we expected (H2) the emergence of SIFI to be especially pronounced under high attentional load. In this case, we might be able to conclude that SIFI can be influenced by attention mechanisms. Conversely, if the emergence of SIFI remains unaffected by attentional load levels, we may infer that SIFI took place during an early and pre-attentive stage. Finally, (H3) to confirm that spatial processing can be asymmetrically influenced by levels of attentional load during multitasking the emergence of SIFI under high attentional load should be more evident for left-sided stimuli compared to right-sided stimuli (i.e., rightward attentional bias).

The rationale of the project has been preregistered beforehand at: https://osf.io/enpjt/?view_only=892930ac0c9a40da96aefc09702c49c7 

## Methods

### Participants and experimental protocol

Participants were recruited by word of mouth among the acquaintances of the research laboratory members, were unaware of the aims and hypothesis of the experiments and received no compensation. Initially, participants were sent an email containing comprehensive instructions and a link to complete the experiments. Upon accessing this link, they were firstly presented with the informed consent form, and only those who clicked the designated button to provide explicit informed consent were able to proceed. Informed consent was obtained from all participants and/or their legal guardians. After that, they encountered the following list of inclusion criteria: age between 18 and 40 years, absence of neurological disorders, absence of severe vision impairments or any other severe medical condition that would prevent from carrying out tasks on the computer and no substance abuse. Only those who confirmed their compliance with the entire list were allowed to go ahead. Subsequently, to prevent potential confounding effects related to handedness, they were asked to respond to an online adaptation of the Edinburgh Handedness Inventory^44^, and only those who were identified as right-handed could continue. At this stage, participants were finally required to execute a practice session and the actual testing phase (see sections below for a detailed description of the testing phase for each experiment). A total of 101 volunteers completed the testing phase of Experiment 1 (mean age: 24.14, age range: 18–34; biological sex: 30 males and 71 females) and a total of 98 volunteers completed the testing phase of Experiment 2 (mean age: 22.63, age range: 18–40; biological sex: 34 males, 63 females, and 1 participant who did not disclose their sex). Importantly, to prevent potential confounding effect related to learning, none of the participants took part in both experiments. Moreover, in Experiment 1, to prevent potential confounding effects related to the hand used to respond, 54 participants responded to lateralized stimuli with their dominant hand, while the remaining participants used their non-dominant hand. The same approach was followed in Experiment 2, where 49 participants used their dominant hand, and the rest used their non-dominant hand (for an analysis of the presence of these confounds see Supplementary information section). Note that the sample sizes were established through a power analysis using the “simr” package (in R Statistical Software, https://cran.r-project.org/doc/FAQ/R-FAQ.html#Citing-R^45^), which leverages on Monte Carlo simulations to calculate power estimates for mixed models^46^. This analysis focused on the power required by statistical tests concerning the two more complex hypotheses, namely H2 and H3 (see analysis section for a detailed description of these tests). We conducted the analysis using as input the preliminary data from a pilot experiment completed by 49 volunteers (mean age: 24.78, age range: 21–33; biological sex: 14 males, 35 females). In this initial experiment the load variation was offline and responses relative to the primary task were given with two distinct keys (“B” for one flash and “N” for two flashes) instead of a single key, generating a Simon effect. Additionally, in the power analysis, we specified an effect size of interest, that was the minimum effect size for the effect to be considered relevant. This effect size was a semi-partial R^2^ of 0.01^47^ that is usually indicative of a small effect^48^. The analysis revealed that 60 participants would suffice to attain a 90% power level for the statistical tests concerning H2 and H3. Nonetheless, given the likelihood of discarding numerous observations in an online setting, we opted to recruit additional participants in both Experiment 1 and Experiment 2. Once finished the testing phase, participants were asked to respond to a questionnaire, designed by the experimenters, to assess user autonomy and test usability. Specifically, they had to select one or more of the following options: “I carried out the experiment to the best of my ability”, “I was interrupted or distracted while carrying out the experiment”, “I completed the experiment quickly without focusing on the given answers”, “I completed the experiment autonomously”, “A person opened the email, and then I completed the experiment autonomously”, “I completed the experiment autonomously, but a person helped me with the use of the mouse and keyboard”, “I completed the experiment with a person who repeated the instructions to me”, “I completed the experiment with a person who suggested a few answers to me”.

The experimental protocol was approved by the Ethics Committee for Psychological Research of the University of Padua (protocol code 3824, date of approval 04/11/2020) and was conducted according to the principles expressed in the Declaration of Helsinki.

#### Experiment 1: dual-task with “offline” manipulation of attentional load

In Experiment 1 the attentional load manipulation was implemented combining the primary audiovisual integration task with a concurrent secondary verbal or spatial working memory task.

**Fig. 1:**
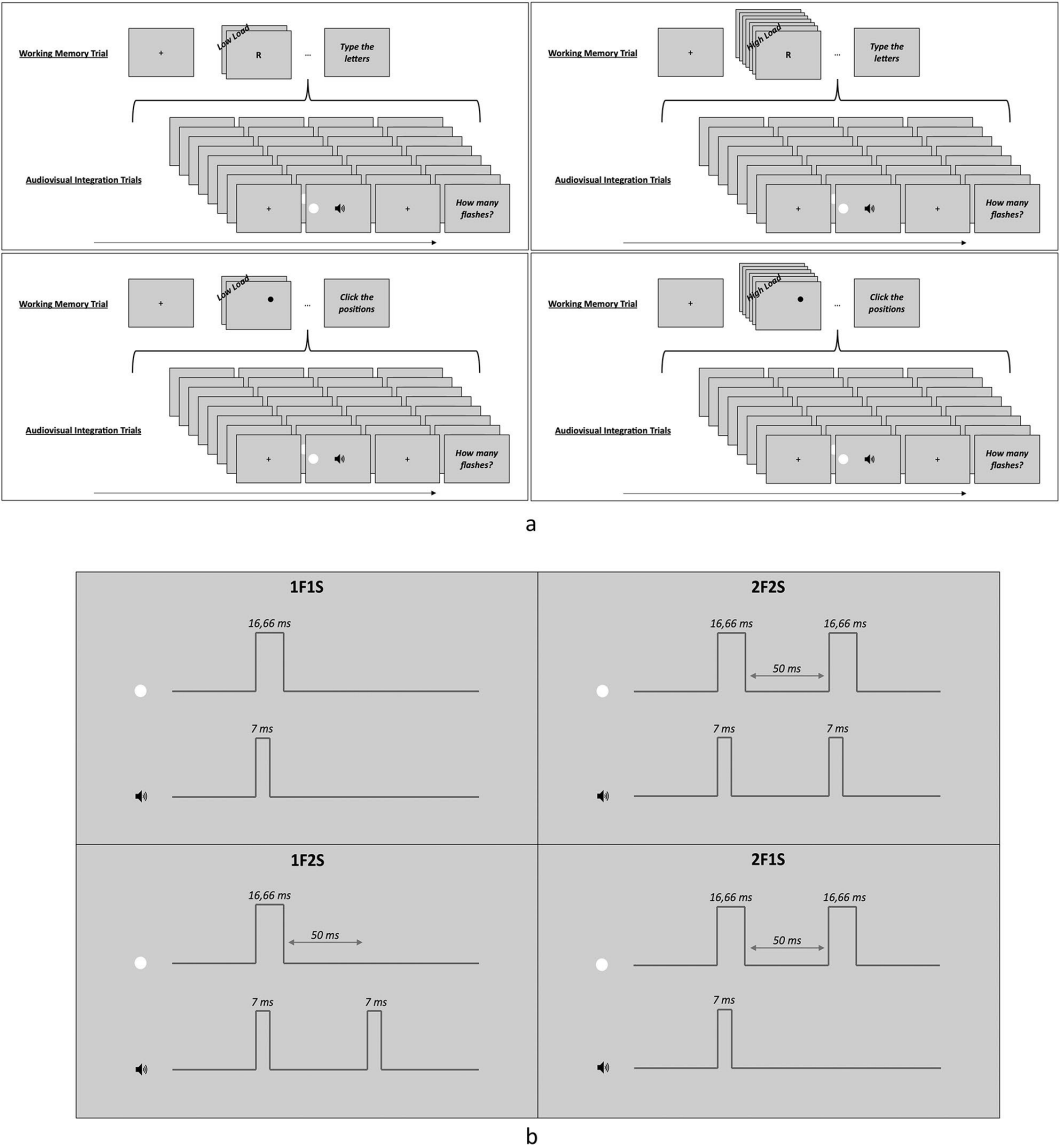
Panel (**a**): Dual-task with “offline” manipulation of attentional load. The figure shows examples of macro-trials, each consisting of a working memory trial and a block of audiovisual integration trials. The working memory trials represent different load conditions: verbal-low load (top-left panel), where participants memorize and report two consonants; verbal-high load (top-right panel), where they memorize and report seven consonants; spatial-low load (bottom-left panel), where they memorize and report two dot positions; and spatial-high load (bottom-right panel), where they memorize and report six dot positions. In all the examples provided, the audiovisual integration trial reported corresponds to the 1F1S-left condition, requiring participants to report the number of flashes when presented with one flash here on the left + one sound. Panel: (**b**) Audiovisual integration trials. During each audiovisual integration trial, participants firstly were presented with lateralized flashes (lasting 16.66 ms) and binaural sounds (lasting 7 ms). The presentation varied based on the condition: either one flash (1F) or two flashes (2F) were presented on the left or on the right side of the screen. In instances of two flashes there was a time lag between the first and second flash lasting for 50 ms. Additionally, depending on the condition, either one sound (1S) or two sounds (2S) were presented, in conjunction with the first flash or in conjunction with both the first and the second flash. Thus, four different audiovisual integration stimulus types were presented, with flashes appearing on the left or on the right side of the screen.

Participants were required to complete *macro-trials*, which consisted in a combination of the two types of trials. Specifically, these macro-trials were formed by a single working memory trial followed by a series of audiovisual integration trials, structured as follows: encoding phase of the working memory trial, audiovisual integration trials and recall phase of the working memory trial. Participants were required to answer to audiovisual integration stimuli after each audiovisual integration trial and to report working memory stimuli at the end of each macro-trial (see Fig. 1, panel a).

##### Audiovisual integration task

During each audiovisual integration trial, participants firstly were presented with a black fixation cross measuring 0.5° and lasting for 500 ms. This initial screen was followed by lateralized flashes and binaural sounds. The lateralized flashes consisted in white discs with diameter measuring 4° and lasting for 16.66 ms (i.e., a refresh rate cycle in commonly used 60 Hz monitors). Depending on the condition, either one flash (1F) or two flashes (2F) were presented on the left or on the right side of the screen with an eccentricity of 8°. In instances of two flashes there was a time lag between the first and second flash lasting for 50 ms. The binaural sounds consisted in hamming windowed sine waveform tones at the frequency of 3.5 kHz and with a duration of 7 ms. Depending on the condition, either one sound (1S) or two sounds (2S) were presented, in conjunction with the first flash or in conjunction with both the first and the second flash. Thus, four different audiovisual integration stimulus types were presented with flashes appearing on the left or on the right side of the screen: 1F1S-left (one flash on the left + one sound), 2F2S-left, 1F2S-left, 2F1S-left, 1F1S-right, 2F2S-right, 1F2S-right and 2F1S-right (see Fig. 1, panel b). During each audiovisual integration trial, participants were required to focus their attention on the flashes only, while ignoring sounds. They were asked to report the number of perceived flashes, by pressing the space bar once for one flash and twice for two flashes with the index finger (of the dominant or non-dominant hand, depending on the counterbalancing). As soon as participants provided their response, the next audiovisual integration trial started.

##### Working memory task

During the encoding phase of each working memory trial, participants were presented with sequences of verbal or spatial stimuli. In the case of verbal attentional load, they were shown a sequence of two consonants (low attentional load) or seven consonants (high attentional load) appearing one after the other. These consonants were written in black, with an Arial font of 50 pixels in advance width, and were positioned in the center of the screen. In case of spatial attentional load participants were presented with a sequence of two dots (low attentional load) or six dots (high attentional load) appearing one after the other. The dots were drawn in black, with a diameter of 1°, and were randomly located on the screen. Each consonant or dot was preceded by a grey screen and lasted for 2000 ms. None of the consonant identities or dot positions were repeated within a sequence. The specific number, size, and duration of the stimuli were all adjusted based on preliminary data previously collected in pilot experiments. Thus, two attentional load types were presented in combination with two attentional load levels: verbal-low load, verbal-high load, spatial-low load and spatial-high load. During the encoding phase of each working memory trial, thus before performing the series of audiovisual integration trials, participants were required to memorize consonant identities or dot positions. During the recall phase, after having performed the series of audiovisual integration trials, they were then asked to report them, by pressing the keyboard or clicking on the screen with the mouse. In both cases responses were considered correct only when stimuli were reported in the exact order in which they were presented (tolerance for the spatial task: 100 pixels from the original dot center). As soon as participants provided their response, the next working memory trial started.

##### Dual-task procedure

The dual-task consisted of 8 blocks, formed by 5 macro-trials. Each macro-trial was composed by one working memory trial and by a series of 16 audiovisual integration trials, organized as follows: encoding phase of the working memory trial, 16 audiovisual integration trials and recall phase of the working memory trial. In total there were 10 trials for each of the four working memory conditions (i.e., verbal-low load, verbal-high load, spatial-low load and spatial-high load), presented in random ordered alternated block by block. As for the audiovisual integration trials, there were 80 trials for each of the eight audiovisual integration conditions (i.e., 1F1S-left, 2F2S-left, 1F2S-left, 2F1S-left, 1F1S-right, 2F2S-right, 1F2S-right and 2F1S-right), presented in random order. Before the testing phase it was conducted a practice session that consisted of 2 macro-trials, one under the verbal-low load condition and one under the spatial-high load condition.

#### Experiment 2: dual-task with “online” manipulation of attentional load

**Fig. 2:**
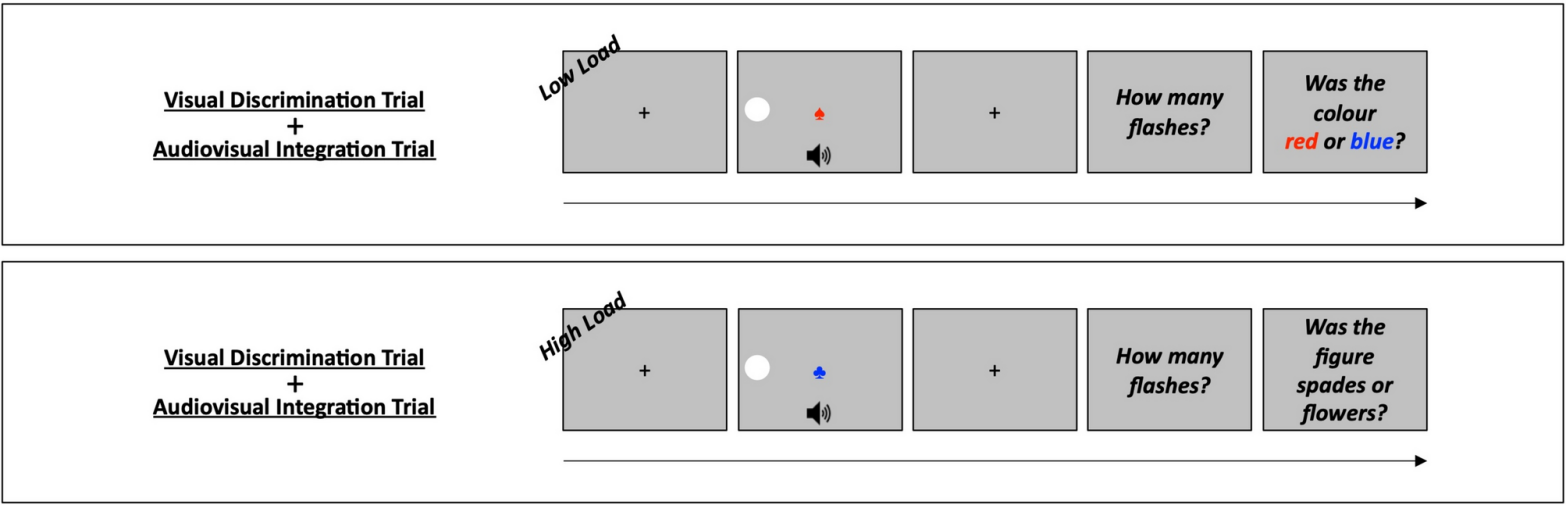
Dual-task with “online” manipulation of attentional load. The figure shows examples of macro-trials, each consisting of an audiovisual integration trial and a visual discrimination trial. In both the example provided, the audiovisual integration trial reported corresponds to the 1F1S-left condition, requiring participants to report the number of flashes when presented with one flash here on the left + one sound. The visual discrimination trials represent different load conditions: low load, where participants discriminate the colour of the central symbol; high load, where they discriminate the shape of the central symbol.

In Experiment 2 the attentional load manipulation was implemented combining the same primary audiovisual integration task described for Experiment 1 with a new concurrent secondary visual discrimination task. Also in this case, participants were required to complete *macro-trials,* which consisted in a combination of the two types of trials. However, differently from Experiment 1, these macro-trials were formed by an audiovisual integration trial and a visual discrimination trial integrated together, with all the stimuli displayed simultaneously. After each macro-trial, participants were required to answer firstly to audiovisual integration stimuli and then to visual discrimination stimuli (see Fig. 2).

##### Audiovisual integration task

Identical to the one used in Experiment 1.

##### Visual discrimination task

While participants were presented with lateralized flashes and binaural sounds, they were also presented with a central symbol consisting in a blue or red spade or flower shape. These symbols were written with an Arial font of 18 pixels in advance width and lasted for 50 ms. The size and duration of the stimuli were all adjusted based on preliminary data previously collected in pilot experiments. During each visual discrimination trial, participants were required to focus their attention either on the colour of the central symbol (i.e., blue vs red), which is an easier task as the colour pops up (low attentional load), or on the shape of the central symbol (i.e., spade vs flower), which is a more difficult and resource consuming task (high attentional load). They were asked to report the colour or the shape, by pressing either the “t” or “y” key with any finger of the hand not used during the audiovisual integration task. Thus, in this case, exclusively manipulating the instructions (i.e., without any perceptual difference between trials), two attentional load levels were presented: low load and high load.

##### Dual-task procedure

The dual-task consisted of 4 blocks, formed by 160 macro-trials. Each macro-trial was composed by one audiovisual integration trial and by one visual discrimination trial, organized as follows: participants firstly were presented with a black fixation cross measuring 0.5° and lasting for 500 ms, followed by audiovisual integration stimuli (i.e., lateralized flashes and binaural sounds) together with visual discrimination stimuli (i.e., central symbol). After each macro-trial, participants were required to answer firstly to audiovisual integration stimuli and then to visual discrimination stimuli. As soon as participants provided both their responses, the next macro-trial started. In total, there were 320 trials for each of the two visual discrimination conditions (i.e., low load and high load), presented in random ordered alternated block by block. As for the audiovisual integration trials, there were 80 trials for each of the eight audiovisual integration conditions (i.e., 1F1S-left, 2F2S-left, 1F2S-left, 2F1S-left, 1F1S-right, 2F2S-right, 1F2S-right and 2F1S-right), presented in random order. Before the testing phase it was conducted a practice session that comprised 64 macro-trials, 32 under the low load condition and 32 under the high load condition.

During both experiments stimuli were presented on a grey background. All responses were collected without time constraints. Instructions were repeated at the start of each block, emphasizing the importance of accuracy over speed. It was also clarified that both tasks were important and that higher scores would only be achieved by correctly answering the secondary tasks. This was to ensure that participants didn’t focus solely on audiovisual integration trials without any attentional load. Participants were allowed to take breaks at the end of each block.

### Materials

Dual-tasks were both programmed using HyperText Markup Language (HTML), Cascading Style Sheets (CSS) and jsPsych, an open-source JavaScript library that provides a flexible framework for building psychological experiments that can be conducted online^49,50^. The dual-tasks were subsequently uploaded to a web server, with JATOS installed, an open-source web application that streamlines the management of web-based data collections. Specifically, JATOS enables hosting experiment scripts, generating links for completing the experiments, and organizing collected data^51^. The web server was located at the Department of General Psychology of the University of Padua. To minimize setting variability, several precautions were taken. Firstly, participants were instructed to complete the task in a semi-dark and quiet room, sitting at a distance of 57 cm from the computer screen. They were advised to keep the computer connected to a power source throughout the testing session to avoid abrupt interruptions. They were indicated to close all internet tabs except for the one related to the experiment, which had to be kept in full-screen mode, without being refreshed, and without going back to previous pages. Additionally, they were asked to avoid using Bluetooth devices for audio output and response recording, as these could potentially introduce delays in stimuli presentation and response collection. When using wired devices, they were directed to align the keyboard’s space bar with the centre of the screen. Furthermore, to ensure consistent stimulus sizes across varying monitor resolutions, jspsych-resize plugin was used. At the outset of each experiment, this tool required participants to use a ruler to adjust the length of a square-shaped container until it measured 10 cm. After that, the ratio between square width in pixels and in millimetres could be calculated, yielding the logical pixel density (LPD) expressed in pixels per mm, which was used to ensure uniform scaling of the size of all stimuli, regardless of the device in use^52,53^. To minimize audio-visual asynchrony, which is one of the less precise aspects of web-based data collections^54^, jsPsych’s capabilities were expanded by utilizing the jspsych-psychophysics plugin^55^. This tool enhanced visual stimuli timing precision (and consequently, ensured highly accurate audio-visual synchronization) by aligning their presentation with the display refresh through the utilization of the requestAnimationFrame method^55^. Finally, to avoid unforeseen issues with the rendering of the experimental webpage, the use of mobile phones or tablets, along with any browser other than Chrome, was blocked.

### Analysis

Data Preparation and data analysis were performed using R Statistical Software, https://cran.r-project.org/doc/FAQ/R-FAQ.html#Citing-R^45^.

#### Data preparation

Firstly, a rigorous data cleaning procedure was implemented to ensure the quality and reliability of the data. To filter out those who might not have provided genuine responses, participants who declared to have been suggested answers or did not provide any feedback in the questionnaire regarding user autonomy and test usability were excluded (n excluded Experiment 1 = 3; n excluded Experiment 2 = 3). Additionally, to filter out those who may have responded way too quickly without due attention or who may have been engaged in other activities during the experiments, participants who completed the experiment in less than 20 min or more than 3 h were also excluded; these cut-offs were determined based on preliminary data previously collected in pilot experiments, where it emerged that experiments would typically take around 50 min for completion (n excluded Experiment 1 = 1; n excluded Experiment 2 = 3). Moreover, to filter out those who might have encountered inconsistent stimuli duration because of technical issues the “avg_frame_time” parameter, provided by the jspsych-psychophysics plugin^55^, was taken into account. This parameter is an indirect measure of the display refresh rate through the use of the requestAnimationFrame method. Theoretically, with a display refresh rate set at 60 Hz, the avg_frame_time would be 16.66 ms. However, in cases where the display operates at a refresh rate different from 60 Hz or when there is a high load during stimulus presentation, the avg_frame_time deviates from 16.66 ms. This variance indicates that, in the specific trial considered, the stimulus may not have been presented with the intended timing. For this reason, participants with individual mean avg_frame_time deviated by more than 1.5 standard deviations from the group’s average avg_frame_time in at least one audiovisual integration stimulus type were excluded (n excluded Experiment 1 = 3; n excluded Experiment 2 = 5). Finally, participants whose individual audiovisual integration accuracy scores deviated by more than 1.5 standard deviations from group’s average audiovisual integration accuracy in at least one audiovisual integration stimulus type were excluded (n excluded Experiment 1 = 9; n excluded Experiment 2 = 8). The same threshold was used for audiovisual integration response times (n excluded Experiment 1 = 0; n excluded Experiment 2 = 0). The same threshold was used also for working memory accuracy in Experiment 1 and visual discrimination accuracy in Experiment 2 (n excluded Experiment 1 = 1; n excluded Experiment 2 = 5) to filter out those who may have not performed at all the secondary task. All criteria were applied to the original samples of participants who completed the experiments; as a result, some participants were excluded due to meeting multiple criteria. The procedure resulted in final samples consisting of 88 participants in Experiment 1 (mean age: 24.36, age range: 18–34; number of males: 28, number of responders with dominant hand: 48) and 80 participants in Experiment 2 (mean age: 22.74, age range: 18–40; number of males: 26, number of responders with dominant hand: 38). Before performing the statistical procedures, individual trials were further pre-processed according to the following steps: trimming avg_frame_time between 14 and 19 ms and trimming audiovisual integration response times between 100 and 4000 ms. These steps resulted in the removal of 1.01% of all initial trials in Experiment 1 and 2.04% of all initial trials in Experiment 2.

#### Models fitting and hypothesises testing

Statistical procedures capitalized on the use of Linear Mixed-effect Models (LMMs) and Generalized Linear Mixed-effect Models (GLMMs)^56^.

##### Secondary task accuracy

To assess the efficacy of secondary tasks in inducing attentional load modulations we initially performed a GLMM (family: binomial; distribution: logit). In Experiment 1 the dependent variable was accuracy in the secondary task. The fixed effects included types (verbal, spatial) and levels (low, high) of attentional load. Participants entered the model as a random intercept, to account for the inherent variations among their baseline accuracy during the secondary task. Additionally, blocks entered the model as a random slope nested within participants; this modelling choice was based on a model selection (see Supplementary information section) and it captured the idea that time on task might have different effects at the individual level. In Experiment 2, the model was identical to the one used in Experiment 1, except for the absence of attentional load types as a fixed effect. In both experiments we expected a main effect of attentional load levels: working memory accuracy (Experiment 1) or visual discrimination accuracy (Experiment 2) would be significantly lower in high attentional load compared to low attentional load. Additionally, in Experiment 1, we explored the potential main effect of attentional load types.

##### Audiovisual integration accuracy

We then conducted a GLMM (family: binomial; distribution: logit) to investigate H1. In Experiment 1 the dependent variable was audiovisual integration accuracy. The fixed effects included: audiovisual integration stimulus types (1F1S, 2F2S, 1F2S, 2F1S), flash presentation side (left, right), attentional load types (verbal, spatial), attentional load levels (low, high) and hand used to respond (dominant, non-dominant). Random effects included also in this case participants as a random intercept and blocks as a random slope nested within participants. Additionally, random effects included audiovisual integration stimulus types as a random slope nested within participants; also this modelling choice aimed at capturing the previously reported individual variability in audiovisual integration accuracy when considering stimuli generating fission and fusion illusions^33^. In Experiment 2, the model was again identical to the one used in Experiment 1, except for the absence of attentional load types as a fixed effect. In both experiments we expected (H1) a main effect of audiovisual integration stimulus types: audiovisual integration accuracy would be significantly lower and SIFI would emerge in incongruent audiovisual integration stimuli, i.e. 1F2S stimuli and 2F1S stimuli, compared to congruent audiovisual integration stimuli, i.e. 1F1S stimuli and 2F2S stimuli.

##### Audiovisual integration d-prime

Finally, we implemented a LMM to investigate H2 and H3. In this case the dependent variable audiovisual integration accuracy was transformed using signal detection theory principles^37,57^, which is a standard approach to investigate sound-induced flash illusion^33^. We aggregated audiovisual integration accuracy values from different congruent and incongruent audiovisual integration stimuli to calculate d-prime (d'). A lower d’ value suggests poorer discrimination ability, that in the present context reflects a greater susceptibility to illusions. Congruent 2F2S stimuli and incongruent 1F2S stimuli were used to calculate d’ related to fission illusion. In 2F2S the correct response “2” was considered a *hit*, while the wrong response “1” was considered a *miss*. In 1F2S stimuli the illusory response “2” was considered a *false alarm*, while the correct response “1” was considered a *correct rejection*. Congruent 1F1S stimuli and incongruent 2F1S stimuli were instead used to calculate d’ related to fusion illusion. In 1F1S stimuli the correct response “1” was considered a *hit*, while the wrong response “2” was considered a *miss*. In 2F1S stimuli the illusory response “1” was considered a *false alarm*, while the correct response “2” was considered a *correct rejection*. From these values, d' was computed as *d'* = *z (hit rate)-z(false allarm rate)*, adjusting for extreme values with the log-linear rule recommended by Hautus^58,59^. In Experiment 1 the so-calculated audiovisual integration d’ entered the LMM as the dependent variable. The fixed effects included: sound-induced flash illusion types (fission, fusion), flash presentation side (left, light), attentional load types (verbal, spatial), attentional load levels (low, high) and hand used to respond (dominant, non-dominant). Random effects included also in this case participants as a random intercept and blocks as a random slope nested within participants. Additionally, random effects included audiovisual integration illusion types as a random slope nested within participants, following the same rationale as the inclusion of audiovisual integration stimulus types as random slope nested within participants in the previous model. In Experiment 2, the model was again identical to the one used in Experiment 1, except for the absence of attentional load types as a fixed effect. In both experiments we expected (H2) a main effect of attentional load levels: the emergence of SIFI would be significantly higher with high attentional load compared to low attentional load. Additionally, in both experiments we expected (H3) an interaction between attentional load levels and flash presentation side: the emergence of SIFI would be significantly higher with high attentional load, especially in case of left-sided flashes.

For all the models in this study, we performed model assumption checks using “DHARMa”^60^. This package employs a simulation-based approach to analyse residuals for fitted LMMs and GLMMs. The analysis indicated small deviations from expected residuals with no evident pattern. None of the models exhibited evident overdispersion, underdispersion, or heteroscedasticity.

To conduct hypothesis tests, LMMs were assessed through Analysis of Variance (Type III) with Satterthwaite’s method for computing degrees of freedom and F statistics^61^, while GLMMs were assessed through Analysis of Deviance with Type III Wald test for computing Chi-square statistics^62^. Post-hoc pairwise comparisons between the levels of fixed factors were tested for main effects and interactions of interest when significant, computing estimated marginal means contrasts^63^ and adjusting for multiple comparisons with false discovery rate^64^.

## Results

We present below the results pertaining to secondary task accuracy, audiovisual integration accuracy, and audiovisual integration d’ for both experiments. We provide details on all the main effects, while we include exclusively those interactions highlighted in the analysis section as relevant for addressing our hypothesis. For each of these main effect and interaction, for what concerns analysis of variance (for LMMs) or analysis of deviance (for GLMMs), we have reported either the F value (for LMMs) or the Chi-square (for GLMMs). For post-hoc pairwise comparisons, we have then reported: the difference between estimated means ($\Delta \widehat{\mu }$, in response scale for LMMs and in log odds ratio scale for GLMMs), as well as, exclusively for GLMMS, the difference between observed means ($\Delta \mu$ in response scale), the standard error (SE) and the associated statistics (*t* test for LMMs and *z* test for GLMMs). Note that odds ratios are the ratios between the frequency with which a correct response occurs in a certain condition and the frequency with which a correct response occurs in another condition.

### Experiment 1

#### Secondary task accuracy

Descriptive statistics for secondary task accuracy (range 0–1) as a function of load types and load levels are summarized in Table 1. The main outputs of the model are summarized in Supplementary information section. Analysis of deviance resulted in the expected main effect of attentional load levels (X^2^ (1) = 297.974, *p* < 0.001), with participants being less accurate in the working memory task in case of high load compared to low load (high vs low: $\Delta \widehat{\mu }$= − 1.7, $\Delta \mu$ = − 0.186, SE = 0.098, *z* = − 17.262, *p* < 0.001). Additionally, we found a main effect of attentional load types (X^2^ (1) = 190.261, *p* < 0.001), with participants being more accurate in the working memory task in case of verbal compared to spatial stimuli (verbal vs spatial: $\Delta \widehat{\mu }$= 1.36, $\Delta \mu$ = 0.139, SE = 0.099, *z* = 13.794, *p* < 0.001).

**Table 1:**
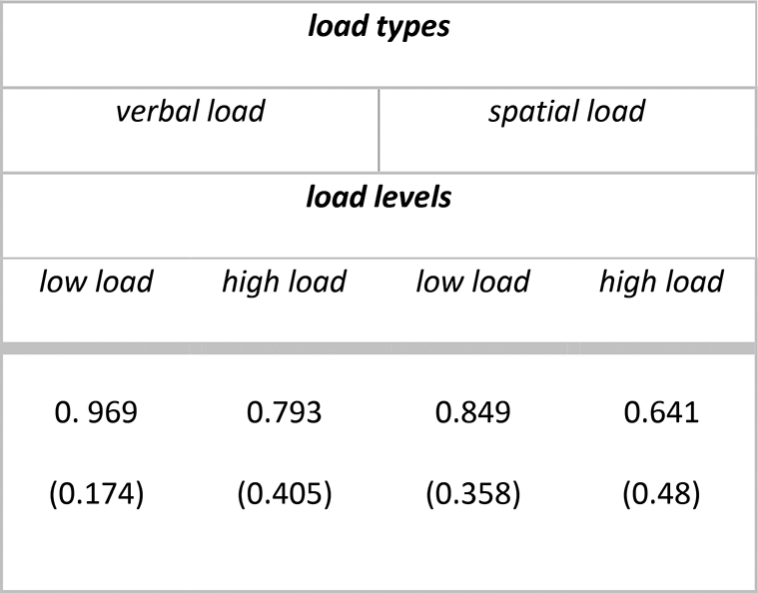
Experiment 1: means (standard deviations) for secondary task accuracy (range 0–1).

#### Audiovisual integration accuracy

**Fig. 3:**
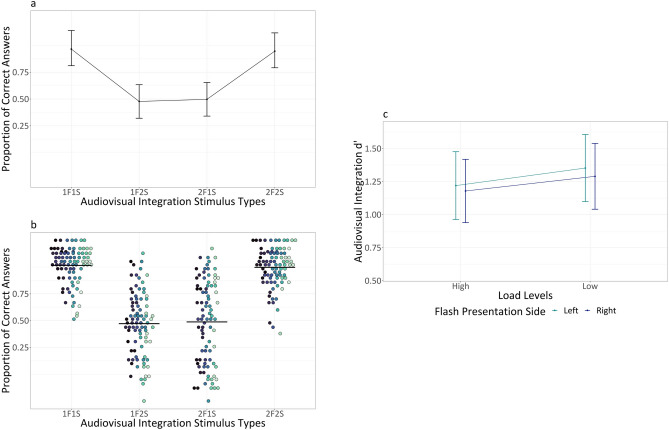
Experiment 1: Panel (**a**) displays averaged proportion of correct answers (i.e., no illusion) for the different audiovisual integration stimulus types. Bars represent 95% confidence intervals, adjusted using the Tryon method as accuracy is a binomial variable. These adjustments were calculated over Anscombe-transformed scores using “superb” (R Package: superb—v 0.9.7.6, Cousineau et al.^65^), and subsequently transformed back into proportions. Panel (**b**) displays individual proportion of correct answers (i.e., no illusion) for the different audiovisual integration stimulus types, with each dot corresponding to a different participant. The group average is indicated by a horizontal black line. All the proportions are depicted using a non-linear scale, specifically the “asn_trans()” scale for arcsine. Panel (**c**) displays cumulative d’ for the different load levels and flash presentation side.

Descriptive statistics for audiovisual integration accuracy (range 0–1) as a function of flash presentation side, audiovisual integration stimulus types, load types and load levels are summarized in Table 2. The main outputs of the model are summarized in Supplementary information section. Analysis of deviance highlighted the expected main effect of audiovisual integration stimulus types (X^2^ (3) = 296.988, *p* < 0.001). Participants were less accurate with incongruent 1F2S stimuli than with congruent 1F1S stimuli (1F2S vs 1F1S: $\Delta \widehat{\mu }$= − 3.413, $\Delta \mu$ = − 0.438, SE = 0.204, *z* = − 16.771, *p* < 0.001) indexing the presence of fission illusion (i.e., due to the delivery of two sounds, two flashes were perceived although only one was presented). Similarly, they were less accurate with incongruent 2F1S stimuli than with congruent 2F2S stimuli (2F1S vs 2F2S: $\Delta \widehat{\mu }$= − 3.137, $\Delta \mu$ = − 0.405, SE = 0.212, *z* = − 14.821, *p* < 0.001) indexing the presence of fusion illusion (i.e., due to the delivery of one sound, only one flash was perceived although two were presented). There were no differences in accuracy between the congruent 1F1S stimuli and 2F2S stimuli (1F1S vs 2F2S: $\Delta \widehat{\mu }$= 0.192, $\Delta \mu$ = 0.011, SE = 0.236, *z* = 0.814, *p* = 0.499) nor between the incongruent 1F2S stimuli and 2F1S stimuli (1F2S vs 2F1S: $\Delta \widehat{\mu }$= − 0.084, $\Delta \mu$ = − 0.022, SE = 0.252, *z* = − 0.335, *p* = 0.738) (see Fig. 3, panel a and b). We can therefore conclude that the main effect of audiovisual integration stimulus types was due to the significant differences between congruent and incongruent stimuli. Moreover, the main effect of flash presentation side was not significant (X^2^ (1) = 2.921, *p* = 0.087), while there was a main effect of attentional load levels (X^2^ (1) = 49.859, *p* < 0.001) and a main effect of attentional load types (X^2^ (1) = 9.312, *p* = 0.002).

**Table 2:**
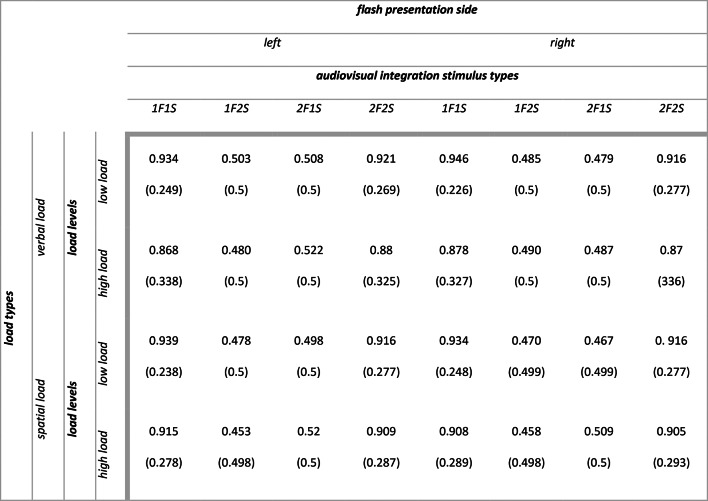
Experiment 1: means (standard deviations) for audiovisual integration accuracy (range 0–1).

#### Audiovisual integration d-prime

Descriptive statistics for audiovisual integration d’ as a function of flash presentation side, audiovisual integration stimulus types, load types and load levels are summarized in Table 3. The main outputs of the model are summarized in Supplementary information section. Analysis of variance highlighted the expected main effect of attentional load levels (F (1, 867.80) = 24.238, *p* < 0.001), with participants having higher illusion rates in case of high load compared to low load (high vs low: $\Delta \widehat{\mu }$= − 0.123, SE: 0.027, *t* (488) = − 4.495, *p* < 0.001). However, the interaction we predicted between attentional load levels and flash presentation side was not significant (F (1, 2175.22) = 0.264, *p* = 0.607) (see Fig. 3, panel c). Moreover, we found a main effect of flash presentation side (F (1, 2175.22) = 6.633, *p* = 0.01) and a main effect of attentional load types (F (1, 1033.60) = 4.499, *p* = 0.034). Resembling audiovisual integration accuracy results, the main effect of sound-induced flash illusion types was not significant (F (1, 86) = 1.541, *p* = 0. 218), as the rates for fission and fusion illusion were similar.

**Table 3:**
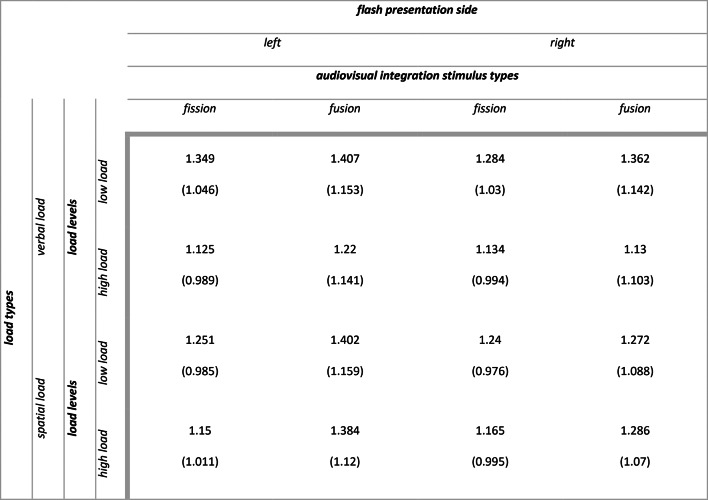
Experiment 1: means (standard deviations) for audiovisual integration d’.

### Experiment 2

#### Secondary task accuracy

Descriptive statistics for secondary task accuracy (range 0–1) as a function of load levels are summarized in Table 4. The main outputs of the model are summarized in Supplementary information section. Analysis of deviance resulted also for Experiment 2 in the expected main effect of attentional load levels (X^2^ (1) = 527.68, *p* < 0.001), with participants being less accurate in the visual discrimination task in case of high load compared to low load (high vs low: $\Delta \widehat{\mu }$= − 2.05, $\Delta \mu$ = − 0.154, SE = 0.089, *z* = − 22.970, *p* < 0.001).

**Table 4:**
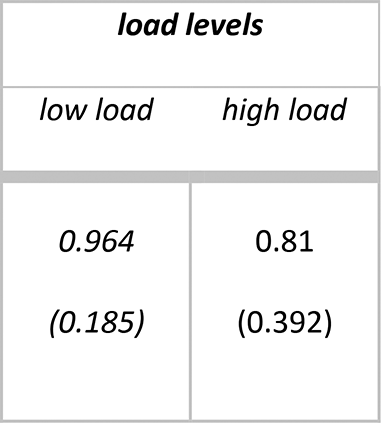
Experiment 2: means (standard deviations) for secondary task accuracy (range 0–1).

#### Audiovisual integration accuracy

**Fig. 4:**
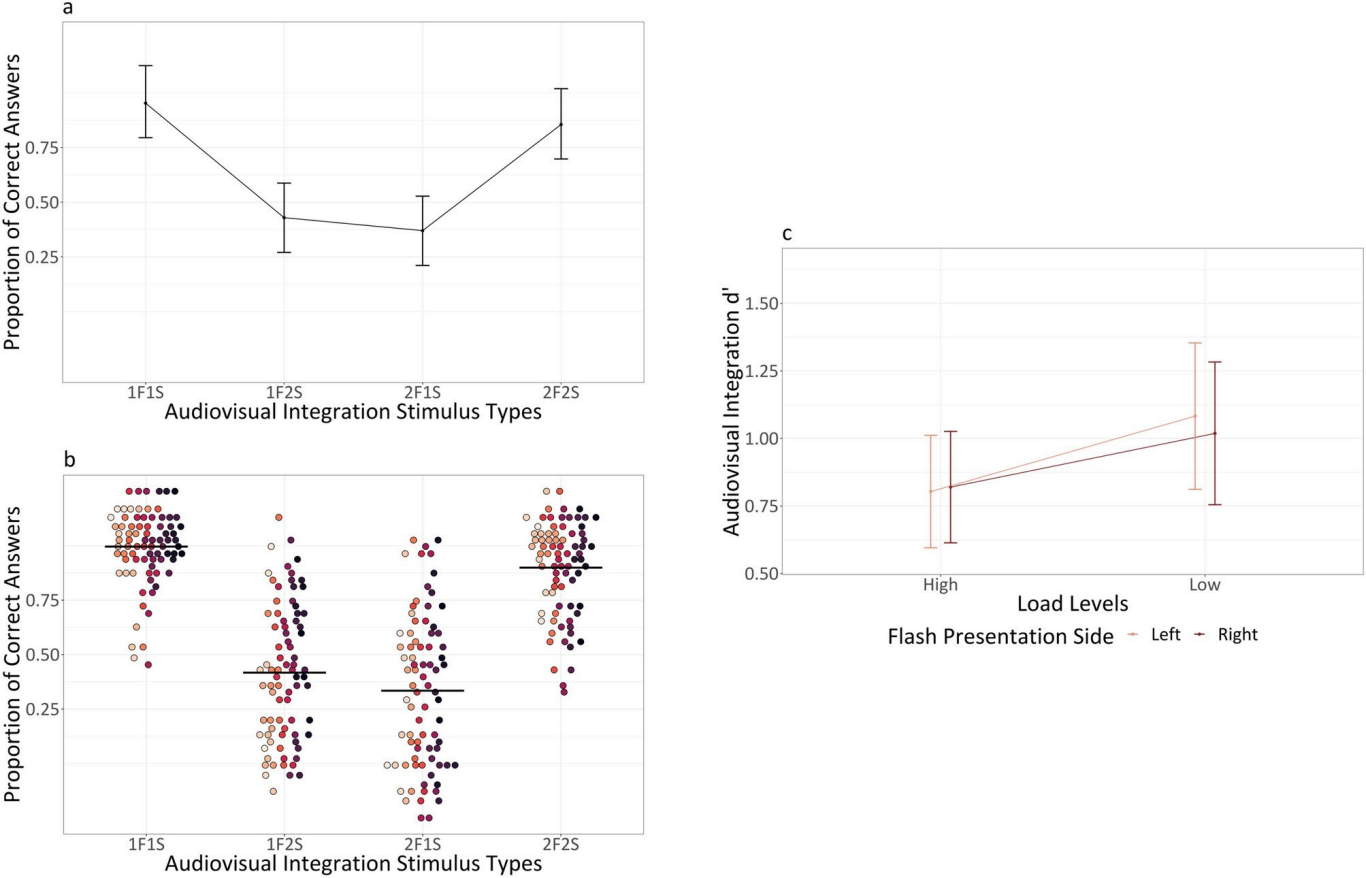
Experiment 2: Panel (**a**) displays averaged proportion of correct answers (i.e., no illusion) for the different audiovisual integration stimulus types. Bars represent 95% confidence intervals, adjusted using the Tryon method as accuracy is a binomial variable. These adjustments were calculated over Anscombe-transformed scores using “superb”^65^, and subsequently transformed back into proportions. Panel (**b**) displays individual proportion of correct answers (i.e., no illusion) for the different audiovisual integration stimulus types, with each dot corresponding to a different participant. The group average is indicated by a horizontal black line. All the proportions are depicted using a non-linear scale, specifically the “asn_trans()” scale for arcsine. Panel (**c**) displays cumulative d’ for the different load levels and flash presentation side.

Descriptive statistics for audiovisual integration accuracy (range 0–1) as a function of flash presentation side, audiovisual integration stimulus types and load types are summarized in Table 5. The main outputs of the model are summarized in Supplementary information section. Analysis of deviance highlighted the expected main effect of audiovisual integration stimulus types (X^2^ (3) = 294.624, *p* < 0.001). Participants were less accurate with incongruent 1F2S stimuli than with congruent 1F1S stimuli (1F2S vs 1F1S: $\Delta \widehat{\mu }$= − 3.344, $\Delta \mu$ = − 0.477, SE = 0.198, *z* = − 16.858, *p* < 0.001) indexing that fission illusion emerged also in this experiment. Additionally, they were less accurate with incongruent 2F1S stimuli than with congruent 2F2S stimuli (2F1S vs 2F2S: $\Delta \widehat{\mu }$= − 3.221, $\Delta \mu$ = − 0.469, SE = 0.242, *z* = − 13.299, *p* < 0.001), indexing that also fusion illusion was reliably detected again. Differently from Experiment 1, there was a significant difference in accuracy between congruent 1F1S stimuli and 2F2S stimuli (1F1S vs 2F2S: $\Delta \widehat{\mu }$= 0.636, $\Delta \mu$ = 0.067, SE = 0.245, *z* = 2.601, *p* = 0.011), but not between incongruent 1F2S stimuli and 2F1S stimuli (1F2S vs 2F1S: $\Delta \widehat{\mu }$ = 0.513, $\Delta \mu$ = 0.059, SE = 0.271, *z* = 1.893, *p* = 0.058) (see Fig. 4, panel a and b). Moreover, the main effect of flash presentation side was not significant (X^2^ (1) = 1.674, *p* = 0.196), while there was a main effect of attentional load levels (X^2^ (1) = 41.07, *p* < 0.001).

**Table 5:**
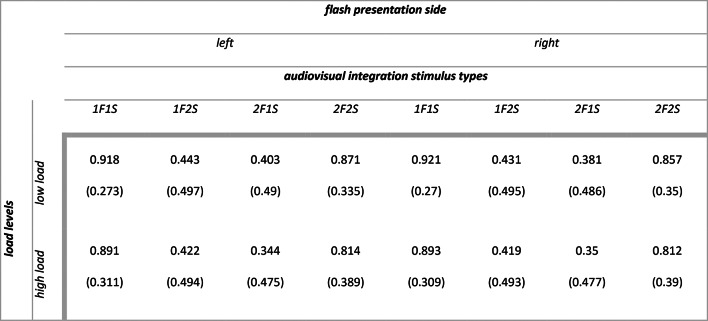
Experiment 2: means (standard deviations) for audiovisual integration accuracy range (0–1).

#### Audiovisual integration d-prime

Descriptive statistics for audiovisual integration d’ as a function of flash presentation side, audiovisual integration stimulus types and load types are summarized in Table 6. The main outputs of the model are summarized in Supplementary information section. Analysis of variance highlighted the expected main effect of attentional load levels (F (1, 193.57) = 34.86, *p* < 0.001), with participants having higher illusion rates in case of high load compared to low load (high vs low: $\Delta \widehat{\mu }$ = − 0.231, SE: 0.041, *t* (194) = − 5.619, *p* < 0.001). Also in Experiment 2 the interaction between attentional load levels and flash presentation side was not significant (F (1, 870) = 1.908, *p* = 0.167) (see Fig. 4, panel c). Resembling audiovisual integration accuracy results, the main effect of flash presentation side was not significant (F (1, 870) = 0.699, *p* = 0. 403) as well as the one of sound-induced flash illusion types (F (1, 78) = 0.383, *p* = 0. 537) meaning similar fission and fusion illusion rates.

**Table 6:**
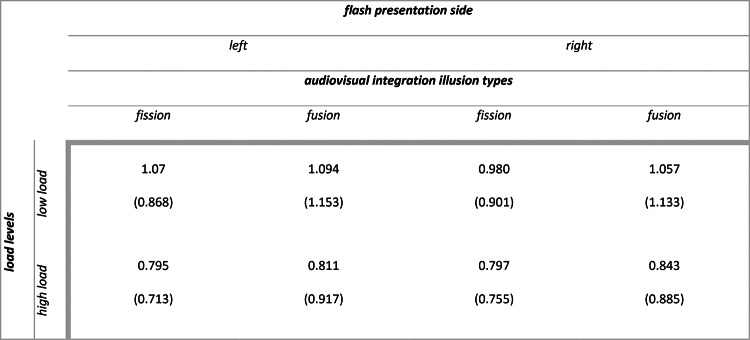
Experiment 2: means (standard deviations) for audiovisual integration d’.

In conclusion, in both experiments, secondary task accuracy was significantly lower under high attentional load compared to low attentional load. Crucially, in both experiments, H1 was confirmed: audiovisual integration accuracy was significantly lower and both fission and fusion illusions emerged in incongruent audiovisual integration stimuli, i.e. 1F2S stimuli and 2F1S stimuli, but not in congruent audiovisual integration stimuli, i.e. 1F1S stimuli and 2F2S stimuli. Moreover, in both experiments, also H2 was verified: the occurrence of SIFI significantly increased under high attentional load. However, H3 was not supported in either experiment: neither of the two different load manipulations revealed that emergence of SIFI under high load was asymmetric across space.

## Discussion

We conducted two experiments to investigate whether levels of attentional load can asymmetrically impact spatial processing in the unimpaired cognitive system during multitasking. In both experiments, we employed a primary audiovisual integration task, characterized by briefly presented stimuli inducing the SIFI on either the left or right side of the screen, along with concurrent secondary tasks that required additional processing either in a more or a less demanding condition. In Experiment 1, the secondary task was a verbal or spatial working memory task, and it allowed us to manipulate attentional load *offline*, meaning that the stimuli used to vary load were presented before the lateralized audiovisual integration stimuli. In Experiment 2, instead, the secondary task consisted in a visual discrimination task, and it enabled us to manipulate attentional load *online*, as the stimuli used to vary the load were presented simultaneously with the lateralized audiovisual integration stimuli. The reason for implementing different secondary tasks in Experiment 1 and Experiment 2 was based on the idea that, assuming the engagement of relatively unspecific attentional resources, the impact on SIFI would have been similar for the different tasks.

For both experiments, we had the following predictions: (H1) audiovisual integration accuracy would be significantly lower and SIFI would emerge in incongruent audiovisual integration stimuli, namely 1F2S stimuli and 2F1S stimuli, (H2) the emergence of SIFI would significantly increase under high attentional load, (H3) especially in case of left-sided flashes.

As a first finding characterizing both experiments, secondary task accuracy was significantly lower when participants were under high attentional load. In Experiment 1, participants exhibited greater accuracy with verbal working memory stimuli compared to spatial working memory stimuli, yet the impact of load was consistently observed in both cases. Therefore, in both experiments, it was possible to confirm the expected effectiveness of secondary tasks in inducing attentional load variations.

In both experiments H1 was clearly confirmed: audiovisual integration accuracy was significantly lower and SIFI emerged in incongruent audiovisual integration stimuli, i.e. 1F2S stimuli and 2F1S stimuli. As expected, 1F2S stimuli successfully triggered the fission illusion whereby participants were presented with one flash, but due to the presence of two sounds, in most of the trials they were overwhelmed by the illusion and tended to split what visually perceived, reporting that they had seen two flashes. Differently, 2F1S stimuli induced the fusion illusion: in this case, participants were presented with two flashes, but due to the presence of a single sound, in most of the trials they were overwhelmed by the illusion and tended to merge what visually perceived, reporting that they had seen a single flash. This result indicates that we were able to successfully replicate SIFI, despite the inherent constraints and drawbacks typically associated with web-based data collections. Since the use of web-based data collections in experimental psychology became mainstream, many effects observed inside the lab have been replicated also in larger samples online^66–72^, including multisensory integration phenomena such as the ventriloquist aftereffect^73^ and the McGurk effect^74^. Our finding contributes to this growing body of evidence, speaking in favour of the reliability of online settings in eliciting effects which are indistinguishable from those obtained in traditional laboratory settings. Additionally, this result indicates that the primary audiovisual integration task was very effective in eliciting to a similar, rather high, extent, not only the ubiquitous fission illusion but also the less commonly reported fusion illusion^75^.

In both experiments, H2 was also verified: the occurrence of SIFI significantly increased under high attentional load. This occurred despite the very different nature of the manipulations supporting the idea that the quantity of attentional resources, rather than their nature, plays a critical role even in seemingly automatic and effortless tasks. Before delving into the potential explanations for this result, it seems important to highlight the complexity of the multifaceted and situation-dependent interplay between multisensory integration and attentional mechanisms, that leads to the experience of the environment around us^76,77^. Some studies support the idea that the binding of multisensory stimuli might be mediated by top-down attentional mechanisms^78,79^. Other studies suggest instead that the binding of multisensory stimuli takes place in an early, pre-attentive stage thanks to bottom-up information^80,81^. More likely multisensory integration is shaped by a combination of the two, together with several other factors^76,77,82^. Within this context, studies that applied dual-tasks to directly investigate how multisensory integration is influenced by attentional load levels yielded different findings. For example, when attentional load was increased during the spatial integration of visuo-tactile stimuli or during the integration of visual cues with emotional valence in songs, there was no significant modulation of multisensory perception^83,84^. Conversely, when attentional load was increased during the processing of audiovisual speech stimuli, illusory multisensory perception was diminished^85,86^. To round out this picture, and in line with our finding, Michail and colleagues^38^ discovered that when attentional load was augmented during the processing of non-speech audiovisual stimuli generating SIFI, illusory perception was actually increased^38,39^. There are multiple, equally compelling alternative explanations for this finding. While it may be challenging to definitively choose among them, they all offer informative and complementary insights into the mechanisms that contribute to the phenomenon. Firstly, according to Michail and colleagues^38^, one possible explanation for the increased SIFI rates under high attentional load could be related to the concept of the temporal window of integration (TWI), which refers to the maximum temporal asynchrony between two different sensory events that allows their perceptual binding into a singular percept^87^. The TWI is known to vary among individuals, and these individual differences can predict susceptibility to SIFI: individuals with narrower TWI can differentiate between audiovisual stimuli that are asynchronous but closely timed and thus are less prone to experience SIFI^88^. As TWI is also influenced by task-specific demands^89^ increased attentional load may have caused its widening, ultimately resulting in enhanced binding of the audiovisual stimuli and increased SIFI rates^38^. Alternatively, according to Michail and colleagues^38^, the increased SIFI rates under high attentional load might be explained according to the “attentional load theory”^90,91^. This theory posits that when perception is deeply taxed, distractor processing may be reduced, whereas when high-level cognitive functions are heavily engaged, the processing of task-irrelevant information may become less inhibited^90,91^. In both Michail and colleagues’ design and our own, auditory stimuli were less critical than visual ones, as participants were instructed to report the number of flashes; thus, auditory stimuli can, in a sense, be considered distractor stimuli or task-irrelevant stimuli. Consequently, it’s possible that the increased attentional load facilitated auditory stimuli processing, allowing them to exert a greater sensory influence, which may have contributed to increased SIFI rates^38^. In our view, another explanation for the increased SIFI rates under high attentional load could be linked to a reduced ability to inhibit automatic and task-irrelevant responses. This idea draws insights from research on ageing, where it has been found that older adults frequently display a reduced ability to filter out task-irrelevant responses^92,93^, as well as an increased susceptibility to SIFI, that is only in part due to age-related changes in unisensory abilities^34^. Notably, the performance in tasks assessing inhibitory control has been found to predict the one in tasks requiring the processing of audiovisual speech stimuli in older but not in younger adults^94^. Additionally, recent studies proposed that a deficit in inhibitory control may be the mechanism at the base of impairments across both postural stability and the processing of non-speech audiovisual stimuli generating SIFI in older adults^95,96^. Given the evidence suggesting that inhibitory control might be reduced when high-level cognitive functions are heavily engaged^97,98^, it is possible to speculate that the increase in attentional load in our tasks may have mirrored what happened with ageing: a decrease in inhibitory control abilities and, consequently, an increase in SIFI rates. In conclusion, our result indicates that SIFI can be influenced by different levels of attentional load and it challenges the notion of an early and pre-attentive onset of the illusion. Rather, it seems that the emergence of SIFI involves, at some point, the use of domain-general, limited and depletable resources that are taxed by increased attentional load. Expanding on this idea, it is plausible that different tasks are characterized by the common use of relatively unspecific attentional resources, irrespective of the specific demands of each task. When the attentional load increases, this general yet limited pool of resources is depleted, potentially affecting the perception of audiovisual stimuli, and leading to the observed modulation of the SIFI effect. While this explanation may appear simplistic at first glance, it aligns well with the similar effects observed under two very distinct load manipulations: in Experiment 1, participants were tasked with retaining either short or long sequences of verbal or spatial working memory stimuli, while in Experiment 2, they were engaged in the processing of visual discrimination stimuli, subject to both simple and complex instructions. However, an alternative and more modular explanation might also hold. Studies on selective interference suggest that secondary tasks tapping into the same domain as the primary task exert a greater influence on its performance^99–101^. The secondary tasks employed in our experiments may have depleted participants’ resources specifically for the visual modality while they were engaged in completing the primary task (i.e., reporting the number of flashes). As a result, when attentional load was increased, participants may have run out of visual resources to then rely more on relatively intact auditory resources. This shift likely led to a greater influence of auditory inputs on responses in the primary task, ultimately increasing SIFI rates.

Finally, regarding H3 both experiments demonstrated the absence of any spatial asymmetry for SIFI under high load, contrary to the expected increase in SIFI rates for left-sided flashes. This result extends the findings reported in the studies by Lisi and colleagues^20^ and by Dodds and colleagues^21^ to a novel context, indicating the absence of load-induced spatial processing asymmetries in healthy participants. The robustness of this result is further strengthened by its consistent replication across a statistically powered sample size and very different load manipulations. The absence of asymmetries cannot be attributed to a floor effect, as the load modulation was clearly present yet manifested itself symmetrically. Previous research that examined the impact of spatial versus non-spatial task instructions^102,103^ emphasized the emergence of asymmetries when spatial aspects are made explicit. For example, Vuilleumier and Rafal^102^, demonstrated that patients with right hemisphere lesions were able to perceive contralesional stimuli when their task involved enumeration, yet they failed to detect the same stimuli when tasked with locating them. Similarly, Cocchini and colleagues^103^, observed that a patient with right hemisphere lesions exhibited extinction symptoms only when required to perform spatial analysis of the stimuli, rather than when simply detecting them^103^. At the same time, however, Peers and colleagues^4^ challenged this view, as they could not find any significant difference between spatial vs non-spatial dual-tasks in patients and also healthy adult groups.

Our result might suggest that the unimpaired and the damaged cognitive systems are not only quantitatively but also qualitatively distinct. Consequently, assessing spatial processing in brain-damaged patients under high attentional load does not exacerbate existing asymmetries in the “baseline system”. Instead, it may be useful to uncover very subtle (yet potentially hazardous) deficits which are almost exclusively contralesional^17^.

This result apparently contradicts the findings reported in the study conducted by Eramudugolla and colleagues^43^, describing load-induced spatial processing asymmetries in the context of processing audiovisual stimuli in healthy participants. However, it is challenging to speculate on the reasons why the results differ between the two studies. This difficulty arises because, despite both studies employed audiovisual stimuli, the audiovisual illusions generated were inherently different: in the study by Eramudugolla and colleagues the stimuli used produced the ventriloquist aftereffect, in which the visual modality guides perception, while in our study, the stimuli generated the SIFI, in which the auditory modality determines what is perceived.

In conclusion, we successfully replicated the increase of sound-induced flash illusion under high attentional load, which challenges the notion of an early and pre-attentive onset of the illusion. However, this effect was not influenced by flash presentation side, which speaks against the existence of load-induced spatial processing asymmetries in the unimpaired cognitive system, at least in the context of audiovisual integration illusions.

## Supplementary Information

Supplementary Information.

## Data Availability

The datasets generated and analysed during the current study are available in the OSF repository, https://osf.io/enpjt/?view_only=892930ac0c9a40da96aefc09702c49c7.
