# Supplementary material for: Attentional load impacts multisensory integration, without leading to spatial processing asymmetries
Source: Sci Rep. 2025 May 9;15:16240. doi: 10.1038/s41598-025-95717-0 (PMC12064717; doi:10.1038/s41598-025-95717-0)
Supplement: Supplementary file 1 — Supplementary Information. [file 41598_2025_95717_MOESM1_ESM.docx]

***ATTENTIONAL LOAD IMPACTS MULTISENSORY INTEGRATION, WITHOUT LEADING TO SPATIAL PROCESSING ASYMMETRIES***

Saccani M.S.^1,2*^, Contemori G. ^3^, Del Popolo Cristaldi F.^3^, Bonato M. ^1,3*^

1. Padova Neuroscience Centre, University of Padua, via Orus 2, 35129, Padua, Italy;

2. IRCCS San Camillo Hospital, via Alberoni 70, 30126, Lido (Venice), Italy.

3. Department of General Psychology, University of Padua, via Venezia 8, 35131, Padua, Italy;

Supplementary Information

Model outputs

The main outputs of each model are summarized in Supplementary Tables 1 – 6.

| ***Predictors*** | *Odds Ratios* | *SE* | *CI* |
| --- | --- | --- | --- |
| *intercept* | 6.86 | 0.56 | 5.86 – 8.05 |
| *load type (verbal vs spatial)* | 0.51 | 0.02 | 0.46 – 0.56 |
| *load level (low vs high)* | 0.43 | 0.02 | 0.39 – 0.47 |
| *Marginal R^2^ =* 0.182; *AIC* = 13823.153 | | | |

**Supplementary Table 1. Experiment 1: main outputs of GLMM for secondary task accuracy.**

| ***Predictors*** | *Odds Ratios* | *SE* | *CI* |
| --- | --- | --- | --- |
| *intercept* | 3.58 | 0.31 | 3.01 – 4.25 |
| *flash presentation side (right vs left)* | 1.02 | 0.01 | 1.00 – 1.05 |
| *audiovisual integration stimulus types (1F2S vs 1F1S)* | 5.66 | 0.98 | 4.04 – 7.94 |
| *audiovisual integration stimulus types (2F1S vs 1F1S)* | 0.19 | 0.03 | 0.14 – 0.24 |
| *audiovisual integration stimulus types (2F2S vs 1F1S)* | 0.20 | 0.04 | 0.14 – 0.29 |
| *load type (verbal vs spatial)* | 1.06 | 0.02 | 1.02 – 1.09 |
| *load level (low vs high)* | 0.88 | 0.02 | 0.85 – 0.91 |
| *Marginal R ^2^* = 0.458; *AIC* = 40431.011 | | | |

**Supplementary Table 2. Experiment 1: main outputs of GLMM for audiovisual integration accuracy~~.~~**

| ***Predictors*** | *Estimates* | *SE* | *CI* |
| --- | --- | --- | --- |
| *intercept* | 1.11 | 0.07 | 0.96 – 1.26 |
| *flash presentation side (right vs left)* | 0.03 | 0.01 | 0.01 – 0.04 |
| *audiovisual integration illusion (fusion vs fission)* | -0.05 | 0.04 | -0.13 – 0.03 |
| *load type (verbal vs spatial)* | 0.03 | 0.01 | 0.00 – 0.05 |
| *load level (low vs high)* | -0.06 | 0.01 | -0.09 – -0.04 |
| *flash presentation side (right) X load level (low)* | -0.01 | 0.01 | -0.02 – 0.01 |
| *Marginal R^2^* = 0.048; *AIC* = 5639.610 | | | |

**Supplementary Table 3. Experiment 1: main outputs of LMM for audiovisual integration d'**

| ***Predictors*** | *Odds Ratios* | *SE* | *CI* |
| --- | --- | --- | --- |
| *intercept* | 16.15 | 1.74 | 13.08 – 19.93 |
| *load level (low vs high)* | 0.36 | 0.02 | 0.33 – 0.39 |
| *Marginal R^2^* *= 0.201; AIC = 28174.809* | | | |

**Supplementary Table 4. Experiment 2: main outputs of GLMM for secondary task accuracy**

| ***Predictors*** | *Odds Ratios* | *SE* | *CI* |
| --- | --- | --- | --- |
| *intercept* | 2.44 | 0.18 | 2.11 – 2.82 |
| *flash presentation side (right vs left)* | 1.02 | 0.01 | 0.99 – 1.05 |
| *audiovisual integration stimulus types (1F2S vs 1F1S)* | 7.09 | 1.15 | 5.16 – 9.75 |
| *audiovisual integration stimulus types (2F1S vs 1F1S)* | 0.25 | 0.04 | 0.18 – 0.34 |
| *audiovisual integration stimulus types (2F2S vs 1F1S)* | 0.15 | 0.03 | 0.10 – 0.22 |
| *load level (low vs high)* | 0.86 | 0.02 | 0.82 – 0.90 |
| *Marginal R^2^* = 0.335; *AIC =* 39269.299 | | | |

**Supplementary Table 5. Experiment 2: main outputs of GLMM for audiovisual integration accuracy~~.~~**

| ***Predictors*** | *Estimates* | *SE* | *CI* |
| --- | --- | --- | --- |
| *intercept* | 0.88 | 0.07 | 0.74 – 1.03 |
| *flash presentation side (right vs left)* | 0.01 | 0.01 | -0.02 – 0.04 |
| *audiovisual integration illusion (fusion vs fission)* | -0.02 | 0.04 | -0.09 – 0.05 |
| *load level (low vs high)* | -0.12 | 0.02 | -0.15 – -0.08 |
| *flash presentation side (right) X load level (low)* | -0.02 | 0.01 | -0.05 – 0.01 |
| *Marginal R^2^* = 0.034; *AIC =* 2589.567 | | | |

**Supplementary Table 6: main outputs of LMM for audiovisual integration d'.**

Model selection for block variable

For each model, a model selection was undertaken to decide how to handle a variable which coded for the block number, independently from the experimental condition characterizing the block. In all cases we compared Akaike's Information Criterion (AIC) and the Bayesian Information Criterion (BIC) of a model “b0” without the block variable, a model “b” with the block variable coded as fixed effect, a model “b1” with the block variable coded as random intercept and a model “b2” with the block variable coded as random slope nested within participants. In both Experiment 1 and Experiment 2, the “b2” model resulted significantly different from other models, with the lowest AIC and BIC values for the GLMM analysing secondary task accuracy, the GLMM assessing audiovisual integration accuracy and also the LMM examining audiovisual integration d' (in all cases *p* < .001). Following this results in all cases the block variable was coded as random slope nested within participants. This modelling choice captured the idea that time on task might have different effects at the individual level. Following this modelling choice any possible effect of increased attentional load should not be influenced by the confounding effect of reduced sustained attention over time on task.

Effect of hand used to respond

In both experiments, in the model with audiovisual integration accuracy and also in the model with audiovisual integration d' as dependent variable, we evaluated the following: 1) presence of a main effect of hand used to respond, to assess whether there was a facilitation in responding with the dominant hand compared to the non-dominant hand 2) presence of an interaction between hand used to respond and flash presentation side, to determine whether there was a stimulus-response compatibility effect, i.e. a facilitation in responding to stimuli presented on the right side with the dominant hand (which was consistently the right hand, as all participants were right-handed) and facilitation in responding to left-sided stimuli with the non-dominant hand (which was consistently the left hand, as all participants were right-handed). In Experiment 1, in the model with audiovisual integration accuracy as dependent variable, analysis of deviance highlighted no effect of hand used to respond (X^2^ (1) = 0.1, *p* = 0.751) as well as no interaction between hand used to respond and flash presentation side (X^2^ (1) = 1.272, *p* = 0.259). In Experiment 1, in the model with audiovisual integration d' as dependent variable, analysis of deviance highlighted no effect of hand used to respond (F (1, 85.21) = 0.017, *p* = 0. 897) as well as no interaction between hand used to respond and flash presentation side (F (1, 2175.22 = 0.38, *p* = 0. 537). In Experiment 2, in the model with audiovisual integration accuracy as dependent variable, analysis of deviance highlighted no effect of hand used to respond (X^2^ (1) = 0.133, *p* = 0.715) as well as no interaction between hand used to respond and flash presentation side (X^2^ (1) = 0.02, *p* = 0.888). Experiment 2, in the model with audiovisual integration d' as dependent variable, analysis of deviance highlighted no effect of hand used to respond (F (1, 77.73) = 0.659, *p* = 0. 419) as well as no interaction between hand used to respond and flash presentation side (F (1, 870) = 0.041, *p* = 0.84).
